# Predictors of *bl*a_CTX-M-15_ in varieties of *Escherichia coli* genotypes from humans in community settings in Mwanza, Tanzania

**DOI:** 10.1186/s12879-016-1527-x

**Published:** 2016-04-29

**Authors:** Stephen E. Mshana, Linda Falgenhauer, Mariam M. Mirambo, Martha F. Mushi, Nyambura Moremi, Rechel Julius, Jeremiah Seni, Can Imirzalioglu, Mecky Matee, Trinad Chakraborty

**Affiliations:** Department of Microbiology/Immunology Weill Bugando School of Medicine, Catholic University of Health and Allied Sciences, BOX 1464, Mwanza, Tanzania; Institute of Medical Microbiology, Justus-Liebig University, Schubertstrasse 81, 35392 Giessen, Germany; Germany and German Center for Infection Research DZIF, Partner site Giessen-Marburg-Langen, Campus Giessen, Giessen, Germany; Department of Microbiology/Immunology, Muhimbili University of Health and Allied Sciences, BOX 65001, Dar es Salaam, Tanzania

## Abstract

**Background:**

Extended-spectrum beta-lactamase (ESBL)-producing Enterobacteriaceae commonly cause infections worldwide. *Bla*_CTX-M-15_ has been commonly detected in hospital isolates in Mwanza, Tanzania. Little is known regarding the faecal carriage of ESBL isolates and *bla*_CTX-M-15_ allele among humans in the community in developing countries.

**Methods:**

A cross-sectional study involving 334 humans from the community settings in Mwanza City was conducted between June and September 2014. Stool specimens were collected and processed to detect ESBL producing enterobacteriaceae. ESBL isolates were confirmed using disc approximation method, commercial ESBL plates and VITEK-2 system. A polymerase chain reaction and sequencing based allele typing for CTX-M ESBL genes was performed to 42 confirmed ESBL isolates followed by whole genome sequence of 25 randomly selected isolates to detect phylogenetic groups, sequence types plasmid replicon types.

**Results:**

Of 334 humans investigated, 55 (16.5 %) were found to carry ESBL-producing bacteria. Age, history of antibiotic use and history of admission were independent factors found to predict ESBL-carriage. The carriage rate of ESBL-producing *Escherichia coli* was significantly higher than that of *Klebsiella pneumoniae* (15.1 % vs. 3.8 %, *p* = 0.026). Of 42 ESBL isolates, 37 (88.1 %) were found to carry the *bla*_CTX-M-15_ allele. Other transferrable resistance genes were *aac(6’)Ib-cr*, *aac(3)-IIa*, *aac(3)-IId*, *aadA1*, *aadA5*, *strA*, *strB* and *qnrS1*. Eight multi-locus sequence types (ST) were detected in 25 *E. coli* isolates subjected to genome sequencing. ST-131 was detected in 6 (24 %), ST-38 in 5 (20 %) and 5 (20 %) clonal complex − 10(ST-617, ST-44) of isolates. The pathogenic phylogenetic groups D and B2 were detected in 8/25 (32 %) and 6/25 (24 %) of isolates respectively. Bla_CTX-M-15_ was found to be located in multiple IncY and IncF plasmids while in 13/25(52 %) of cases it was chromosomally located.

**Conclusion:**

The overlap of multi-drug resistant bacteria and diversity of the genotypes carrying CTX-M-15 in the community and hospitals requires an overall approach that addresses social behaviour and activity, rationalization of the antibiotic stewardship policy and a deeper understanding of the ecological factors that lead to persistence and spread of such alleles.

## Background

Extended-spectrum beta-lactamase (ESBL)-producing Enterobacteriaceae are currently a major problem in hospitalized patients worldwide [1–3]. The prevalence of ESBLs among clinical isolates varies between countries and from institution to institution [2, 4]. Tanzania is one of the sub-Saharan African countries facing increasing numbers of health care associated infections due to multi-drug resistant Gram-negative bacteria [5–8]. Data regarding ESBL isolates in Tanzania are limited to tertiary hospital based studies only.

Several studies performed in developed countries have demonstrated ESBL carriage in the community [9–13]. In addition, in many studies from developed and middle-income countries it could be demonstrated that ESBL-producing bacteria are common in domestic and companion animals in the community [14, 15]. Human to animal contact seems to play a role in the transfer of ESBL producing bacteria between both populations [14, 16, 17]. In Thailand, different factors such as better education, history of hospitalization and the use of antibiotics have been found to be independently predictors for ESBL carriage [18]. Despite the potential risk of ESBL acquisition in the community and transfer between humans and animals there is no study which has documented ESBL carriage and associated risk factors in the human community in Tanzania. Therefore this study was done to investigate the magnitude of ESBL carriage and diversity of ESBL genotypes, and to identify factors associated with it among humans in the community in Mwanza.

## Methods

### Sample size and sampling

A cross-sectional study was conducted between June and September 2014 in three rural districts (Igogo, Mbugani and Kirumba) with squatters in Mwanza city. The characteristics of the rural districts are described in Table 1. Sample size was calculated using the formula by Cochran [19]. A prevalence of 22.1 % from a study performed in Madagascar was used for calculation [20]. The minimum sample size obtained was 297.

**Table 1:** Profile of Igogo, Mbugani and Kirumba

Variables	Igogo	Mbugani	Kirumba
Population size	27,303	39,041	29,354
Number of hospitals/Dispensaries	2	5	4
Number of pharmacy/Medicine shops	18	10	4
Population per pharmacy/medicine shops	1516.8	3904.1	7338.5
Industrial activities	• Industrial areas • Car garages • Storages warehouses	• None	• Car garages
Type of toilets	Latrines	Latrines	Latrines
Damping ground	Present	Present	Present
Distance from Tertiary hospital (Bugando Medical Centre)	1.56 km	2.16 km	3.82 km
ESBL rates	20.5 %	15.2 %	11.6 %

Streets within these rural districts with characteristics of squatter settlements were purposively selected. A total of 3144 households were obtained from these streets. The number of households in each street was obtained from the household registers at the street Executive Officer‘s office. Simple random sampling was used to select 334 households which were included in the study. Using a random number generator, the households to be included in the study were determined. A total of 334 stool samples (one per participant) were collected. From every participant enrolled, additional information (with the use of an interview questionnaire) such as age, gender, size of the family, history of antibiotic use in the past one month and history of admission in the past one year, were collected.

### Laboratory procedures

A total of 334 non-repetitive stool specimens were obtained from humans. All specimens were cultured on MacConkey agar supplemented with 2 μg/mL cefotaxime (Oxoid, Basingstoke, UK) and plain MacConkey agar plates to isolate lactose fermenting colonies to investigate these for the antimicrobial susceptibility pattern.

### Strain selection

One colony from predominant morphologically similar colonies was selected on the MacConkey agar plate with 2 μg/mL cefotaxime for subsequent characterization while a representative of predominant morphologically similar lactose fermenter colonies was also selected from a plain MacConkey agar plate.

### ESBL confirmation and susceptibility testing

ESBL isolates were confirmed using disc approximation method as previously described [5], commercial ESBL CHROMagar (CHROMagar Company, Paris, France) and VITEK-2 compact system (AST-card N214 and N248, bioMérieux, Nürtingen, Germany) in case of ambiguous results. Antimicrobial susceptibility testing using ciprofloxacin (5 μg), gentamicin (10 μg), tetracycline (30 μg) and trimethoprim/sulphamethoxazole (1.25/23.75 μg) was performed with all isolates based on Clinical Laboratory Standard Institute (CLSI) Guidelines [21].

### Analysis of the CTX-M-Allele

A total of 42 ESBL-producing isolates were available for further characterization. First, the presence of CTX-M was identified using CTX-M-F (sequence: 5’-TCTTCCAGAATAAGGAATCCC-3’) and CTX-M-R (sequence: 5’-CCGTTTCCGCTATTACAAAC-3’) amplifying 909 bp of the *bla*_CTX-M_ gene. In case of ambiguities and additional set of primers (CTF-F: 5’-GACAGACTATTCATGTTGTTG-3’ and CTF-R: 5’-CGATTGCGGAAAAGCACGTC-3’) was used to differentiate *bla*_CTX-M-15_ from *bla*_CTX-M-28_. All CTX-M products were sequenced with both forward and reverse primers using the automated sequencer ABI Prism® 3100 (Life technologies, Road Grand Island, USA). The blastN algorithm of NCBI (http://www.ncbi.nlm.nih.gov/blast/) was to identify the ESBL alleles.

SHV-F (5’-GCAAAACGCCGGGTTATTC-3’) and SHV-R (5’-GGTTAGCGTTGCCAGTGCT-3’) were used to amplify 940 bp of the *bla*_SHV_ gene. PCR using the primers TEM-F (5’-ATGAGTATTCAACATTTCCG-3’) and TEM-R (5-TTAATCAGTGAGGCACCTAT-3’) was used to amplify 851 bp of the *bla*_TEM_ gene [22].

### Whole genome sequencing

Twenty five CTX-M-15-producing *Escherichia coli* strains were randomly selected for whole genome sequencing (WGS). Genomic DNA was isolated using Purelink Genome DNA Mini kit (Invitrogen, Darmstadt, Germany) according to the manufacturer’s instruction. WGS was carried out on an Illumina MiSeq instrument (Illumina, San Diego, CA, USA) with an Illumina Nextera XT library with 2x300bp paired-end reads. The reads were assembled using SPAdes (version 3.0) [23]. The assembled contigs were analysed and examined for the presence of transferrable resistance genes, virulence genes, multi-locus sequence types, and plasmid replicon types using ResFinder, VirulenceFinder, MLST 2.0, and PlasmidFinder software, [24–27], respectively.

The location of *bla*_CTX-M-15_ was determined by extracting the contigs harbouring *bla*_CTX-M-15_ and studying the genes flanking *bla*_CTX-M-15_ gene using NCBI blast (http://blast.ncbi.nlm.nih.gov/Blast.cgi).

### Data analysis

Data were double-entered into Microsoft Excel and analysed using STATA version 11. Results were summarized using proportions (%) for categorical data and medians (IQR) for continuous variables. Categorical variables were compared using either Pearson’s Chi–squared or Fisher’s exact test, where appropriate. To determine the predictors of ESBL carriage, univariate followed by multivariate logistic regressions analysis was performed. The predictors tested included age, sex, number of residents in a household, antibiotic use in the last month, admission history and presence of animals at home. Odd ratios with respective 95 % confidence interval (CI) were reported. Predictors with a *p*-value of less than 0.05 were considered statistically significant.

### Limitations of the study

In this study the primers pair used are not for amplification of all CTX-M groups, and might have introduced a great bias towards group 1 to which CTX-M-15 belongs. However, the sequence covered aligned clearly align to the product with CTX-M-15 standard and this was further confirmed for the 25 isolates which underwent WGS. The WGS of 25 randomly selected isolates also confirmed the presence CTX-M-15 in all 25 isolates. Finally, 13 ESBL isolates could not be recovered for PCR and sequencing.

## Results

### Demographic

Of 334 humans sampled, 196 (58.7 %) were female. The median age was ten years (IQR 5–23). All sampled participants used tap water. The median number of children in a household was three (IQR 2–4). The majority of participants 156/334 (46.7 %) were from Igogo rural district (Table 1).

### ESBL carriage and rates of ESBL by isolates

Of 334 individuals from the Mwanza city community, 55 (16.5 %) were found to be colonized by ESBL-producing Enterobacteriaceae. A total of 323 lactose fermenting isolates (270 *E. coli* and 53 *Klebsiella pneumoniae*) were obtained from plain MacConkey agar plates. Out of *E. coli* and *Klebsiella pneumoniae* isolates, 42/270 (15.5 %) and 2/53 (3.8 %) were ESBL producers, respectively (*p* = 0.026). *E. coli* (42/55; 76.3 %) formed the majority of ESBL isolates in this population. A total of eleven ESBL isolates were other Enterobacteriaceae species (*Enterobacter spp: n* = 4*, Proteus mirabilis: n* = 5 *and Proteus vulgaris: n* = 2*)*. Their ESBL rates could not be calculated because these isolates were not targeted for in using plain MacConkey agar plates.

### Susceptibility pattern

A total of 279 (*E. coli,* 228 and *Klebsiella pneumoniae,* 51) non-ESBL isolates and 55 ESBL isolates from humans stool specimens were obtained. The 55 ESBL isolates were significantly more resistant to trimethoprim/sulphamethoxazole (SXT), tetracycline (TET), gentamicin (CN) and ciprofloxacin (CIP) than the non-ESBL isolates; Table 2 (*p* < 0.001). All isolates were sensitive to ertapenem, meropenem, imipenem and tigecycline. The resistance rates of the isolates to TET, SXT, CN and CIP were 48.8 %, 64.9 %, 14.4 % and 12.9 % respectively.

**Table 2:** Resistance rates of ESBL and Non-ESBL isolates to TET, CIP, CN and SXT

Antibiotic	NON-ESBL (*n* = 279)	ESBL (*n* = 55)	Total (*n* = 334)	*P* value
Tetracycline	119 (42.7 %)	44 (80 %)	163 (48.8 %)	*p* < 0.001
Ciprofloxacin	6 (2.1 %)	37 (67.2 %)	43 (12.9 %)	*p* < 0.001
Gentamicin	14 (5.2 %)	34 (61.8 %)	48 (14.4 %)	*p* < 0.001
Co-trimoxazole	184 (66.6 %)	52 (94.5 %)	216 (64.7 %)	*p* < 0.001

*SXT* Trimethoprim/sulphamethoxazole, *TET* tetracycline, *CN* gentamicin and *CIP* ciprofloxacin

### Predictors of ESBL carriage

Higher median age was observed among individuals colonized with ESBLs compared to those without colonization (17 [IQR 6–38] vs. 10 [IQR 5–22], *p* = 0.028). On univariate analysis, the ESBL carriage significantly increased with the number of children in the household (OR = 1.34, 95 % CI 1.14–1.57, *p* < 0.001). Humans from Igogo were significantly more colonized by ESBL-producing isolates than those from Kirumba on univariate analysis (OR = 1.9, 95 % CI 1.0–4.2, *p* = 0.05).

Of 208 individuals who had a history of using antibiotics in the past one month, 44 (21.1 %) were found to be colonized with ESBL isolates as compared to 11 (8.7 %) of those with no history of antibiotic use (OR = 2.88, 95 % CI 1.3–5.66, *p* = 0.004). In addition, significantly higher rate of colonization was observed among individuals with history of admission in the past one year compared to those with no history (66.6 % vs. 15.1 %; *p* = 0.001).

On multivariate logistic regression analysis only history of admission, history of antibiotic use and increasing age were independent predictors of ESBL carriage (Table 3).

**Table 3:** Predictors of ESBL carriage among 334 humans in the community in Mwanza, city

Variables	Positive ESBL carriage (55)	Univariate analysis		Multivariate analysis	
		OR (95 % CI)	*P* value	OR (95 % CI)	*P* value
Age(years)^a^	17 (IQR 6–17)	1.02 (1.2–1.3)	0.028	1.07 (1.04–1.10)	<0.001
Number of children^a^	4 (IQR 2–5)	1.34 (1.14–1.57)	<0.001	1.15 (0.95–1.39)	0.134
Sex					
Male (138)	22 (15.9 %)	1			
Female (196)	33 (16.8 %)	1.06 (0.94–1.72)	0.828	1.33 (0.67–2.63)	0.410
Location					
Kirumba (112)	13 (11.6 %)	1			
Mbugani (66)	10 (15.2 %)	1.35 (0.49–3.59)	0.49		
Igogo (156)	32 (20.5 %)	1.9 (1.0–4.2)	0.05	1.33 (0.901–1.97)	0.149
Antibiotic use					
Yes (208)	44 (21.2 %)	1			
No (126)	11 (8.7 %)	2.8 (1.38–5.6)	0.004	27 (6.63–116)	<0.001
Admission history					
No (325)	49 (15.1 %)	1			
Yes (9)	6 (66.7 %)	11.3 (2.7–46.5)	0.001	7.4 (1.43–38.5)	0.017

^a^median

### ESBL alleles and resistance genes

Of 42 ESBL isolates available for typing, 37 (88.1 %) were found to carry the *bla*_CTX-M-15_ allele. Of these, 34 (91.8 %) were *E. coli*, two *Klebsiella pneumoniae* and one *Enterobacter* spp. The remaining five ESBL isolates (three *E. coli* isolates and two *Enterobacter* spp.) were not positive for CTX-M group 1 alleles. Further screening for the presence of SHV- and TEM-type ESBL-genes was negative. The whole genome sequence of 25 randomly selected E. coli strains confirmed all to harbour *bla*_CTX-M-15_. Analysis of the sequenced isolates revealed that in 13/25 isolates CTX-M-15 was found to be located in the chromosome.

Several other transferrable resistance genes were detected in the sequenced *E. coli* genomes. Aminoglycoside resistance genes detected were *str*A/*str*B (18/25, 72 %), *aadA5* (16/25, 64 %), *aac(6’)Ib-cr* (17/25, 68 %), *aac(3)-IIa* (12/25, 48 %), *aadA1* (5/25, 20 %) and *aac(3)-IId* (6/25, 24 %). Quinolone resistance genes detected were *aac(6’)Ib-cr* (17/25, 68 %) and *qnrS1* (6/25, 24 %) (Table 4). All sequenced isolates carried trimethoprim and sulphamethoxazole resistance genes. All but two isolates harboured tetracycline resistance genes.

**Table 4:** Resistance genes, sequence types and plasmid replicons of the sequenced isolates

Isolate	Beta-Lactam genes	Other antibiotic resistance genes	Plasmid replicon type	pMLST	Sequence type	Phylogenetic group
RA005	^*a*^ *bla*_CTX-M-15_, *bla*_OXA-1_, *bla*_TEM-1B_	*aadA5, aac(6’)Ib-cr, aac(3)-IIa*	IncFIA, IncFIB, IncFII	F1:A1:B1	ST-648	D
RA023	^*a*^ *bla*_CTX-M-15_	*aadA5, qnrS1*	IncY	-	ST-4450	A
RA025	^*a*^ *bla*_CTX-M-15_	*aadA5, qnrS1*	IncY	-	ST-4450	A
RA034	^*a*^ *bla*_CTX-M-15_, *bla*_OXA-1_, *bla*_TEM-1B_	*aadA5, aac(6’)Ib-cr, aac(3)-IIa*	IncFIA, IncFIB, IncFII	F1:A1:B1	ST-648	D
RA043	*bla*_CTX-M-15_ *, bla*_TEM-1B_	*strB, strA, qnrS1*	IncY	-	ST-2852	B1
RA045	^*a*^ *bla*_CTX-M-15_, *bla*_OXA-1_ *bla*_TEM-1B_	*aadA5, aac(6’)Ib-cr, aac(3)-IIa*	IncFIA, IncFIB, IncFII	F1:A1:B1	ST-648	D
RA051	*bla*_CTX-M-15_, *bla*_OXA-1_, *bla*_TEM-1B_	*aadA5,aac(6’)Ib-cr, aac(3)-IIa, strA, strB*	IncFIA, IncFIB, IncFII	F31:A4:B1	ST-617	A
RA061	^*a*^ *bla*_CTX-M-15_, *bla*_OXA-1_	aadA1, aac(6’)Ib-cr, aac(3)-IIa,strA, strB	IncFIB, IncFII	F1:A-:B33	ST-38	D
RA073	^*a*^ *bla*_CTX-M-15_, *bla*_OXA-1_	aadA5, aac(6’)Ib-cr, aac(3)-IIa,strA, strB	IncFIA, IncFIB, IncFII	F1:A1:B16	ST-131	B2
RA085	*bla*_CTX-M-15_, *bla*_TEM-1B_	*strB, strA, qnrS1*	IncFIA, IncFIB, IncFII, IncY	F1:A1:B20	ST-2852	B1
RA102	^*a*^ *bla*_CTX-M-15_, *bla*_OXA-1_	*aadA1, aac(6’)Ib-cr, aac(3)-IIa, strA, strB*	IncFIB, IncFII	F1:A-:B33	ST-38	D
RA105	*bla*_CTX-M-15_, *bla*_OXA-1_, *bla*_TEM-1B_	aadA5, aac(6’)Ib-cr, aac(3)-IId, strA,strB	IncFIA, IncFIB, IncFII	F87:A4:B1	ST-617	A
RA116	^*a*^ *bla*_CTX-M-15_, *bla*_OXA-1_	*aadA5, aac(6’)Ib-cr,aac(3)-IIa,strA, strB,*	IncFIB, IncFIA, IncQ1, IncFII	F1:A1:B16	ST-131	B2
RA134	^*a*^ *bla*_CTX-M-15_	*aadA1, strA, strB*	No replicon		ST-205	B1
RA166	*bla*_CTX-M-15_, *bla*_OXA-1_	*aadA5, aac(6’)Ib-cr, aac(3)-IIa,*	IncFIA, IncFIB,	F31:A4:B1	ST-131	B2
RA173	^*a*^ *bla*_CTX-M-15_, *bla*_OXA-1_, *bla*_TEM-1B_	*aadA5, aac(6’)Ib-cr, aac(3)-IId, strA,strB*	IncFIB, IncFIA, IncQ1, IncFII	F48:A1:B26	ST-131	B2
RA175	^*a*^ *bla*_CTX-M-15_, *bla*_OXA-1_, *bla*_TEM-1B_	*aadA5, aac(6’)Ib-cr, aac(3)-IId, strA, strB*	IncFIA, IncFIB, IncQ1, IncFII	F48:A1:B26	ST-131	B2
RA176	*bla*_CTX-M-15_, *bla*_TEM-1B_	*strB, strA,qnrS1*	IncY	-	ST-38	D
RA194	*bla*_CTX-M-15_, *bla*_OXA-1_, *bla*_TEM-1B_	*aadA5,aac(6’)Ib-cr, aac(3)-IIa, strA, strB*	IncFIA, IncFIB, IncFII	F31:A4:B1	ST-617	A
RA195	*bla*_CTX-M-15_, *bla*_TEM-1B_	*aadA1, aac(3)-IId, strB, strA*	IncFIB, IncFII	F1:A-:B33	ST-38	D
RA217	*bla*_CTX-M-15_, *bla*_OXA-1_	aadA5, aac(6’)Ib-cr,aac(3)-IIa, strA, strB	IncFIA, IncFIB, IncFII	F31:A4:B1	ST-44	A
RA218	*bla*_CTX-M-15_, *bla*_OXA-1_	*aadA5, aac(6’)Ib-cr,aac(3)-IIa, strA, strB*	IncFIB, IncFIA, IncFII	F31:A4:B1	ST-44	A
RA228	^*a*^ *bla*_CTX-M-15_, *bla*_OXA-1_	*aadA1, aac(6’)Ib-cr, aac(3)-IIa,,strA, strB*	IncFIB, IncFII	F1:A-:B33	ST-38	D
RA246	*bla*_CTX-M-15_, *bla*_OXA-1_, *bla*_TEM-1B_	aadA5, aac(6’)Ib-cr, aac(3)-IIa, strA,strB	IncFIA, IncFII	F2:A1:B-	ST-131	B2
RA256	*bla*_CTX-M-15_, *bla*_TEM-1B_	*strB, strA,qnrS1*	IncY, IncFIB	F46:A-:B24	ST-2852	B1

^*a*^in these isolates CTX-M-15 was located in the chromosome

### Multi locus sequence types (MLST), phylogenetic groups and plasmid MLST

Eight different sequence types were observed in the genome-sequenced strains (Table 4). The most common ones were ST131 (6/25, 24 %), ST38 (5/25, 20 %) and ST-648 (3/25, 12 %), ST2852 (3/25, 12 %), and ST617 (3/25, 12 %), Out of 25 isolates, eight (32 %) were members of phylogroup D, seven (28 %) of phylogroup A, six (24 %) of group B2 and four of B1.

**Fig. 1:**
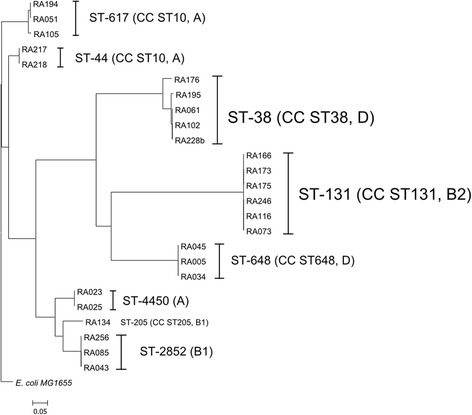
Phylogenetic tree of 25 ESBL-producing *E. coli* based on the whole genome rooted from *E. coli* MG1655 genome. The tree was produced using Harvest Suite and drawn by MEGA5 software

WGS-based phylogenetic tree grouped the isolates into three large clusters, all pathogenic *E. coli* (group B2 and D) were grouped into one cluster with 14 (56 %) isolates. The other two clusters were clonal complex (CC) ST-10 (ST-617, ST-44, all phylogenetic group A) and another cluster with a mixture of phylogroup A (ST-4450) and B1 (ST-2852 and ST-205) (Fig. 1).

A total of 20 (80 %) isolates were found to carry IncF plasmids which were characterized using plasmid-based multi-locus sequence typing (pMLST) to give six different pMLST (F1:A1:B1 (3/25), F31:A4:B1 (5/25), F1:A-:B33 (4/25), F48:A1:B26 (2/25), F1:A1:B16 (2/25), F1:A1:B20, F2:A1:B-, F87:A4:B1 and F46:A-:B24 variants (Table 4).

In most cases, isolates exhibiting an identical sequence type were found to carry different plasmid types.

### Virulence genes

Isolates belonging to the phylogenetic group B2 harboured the most virulence genes followed by phylogroup D isolates. Glutamate decarboxylase (*gad*) that confers resistance to bile salts and the increased serum survival genes (*iss*) were detected in 13 (52 %) of the isolates. All phylogroup B2 isolates harboured the *sat* and *Iha* genes encoding for a serine-protease autotransporter and an iron-dependent adhesion protein, respectively (Table 5).

**Table 5:** Virulence factors in relation to ST and phylogenetic group

Isolate	Sequence type	Phylogroup	Virulence gene
RA005	ST-648	D	*iha, sat, Ipfa, nfaE, gad*
RA023	ST-4450	A	*gad, IpfA*
RA025	ST-4450	A	*gad, IpfA*
RA034	ST-648	D	*iha, sat, Ipfa, nfaE*
RA043	ST-2852	B1	*gad, IpfA*
RA045	ST-648	D	*iha, sat, Ipfa, nfaE*
RA051	ST-617	A	*gad, iss*
RA061	ST-38	D	*gad, iss*
RA073	ST-131	B2	*iha, sat, Ipfa, nfaE, gad, senB*
RA085	ST-2852	B1	*gad, IpfA*
RA102	ST-38	D	*gad, iss*
RA105	ST-617	A	*gad, iss*
RA116	ST-131	B2	*iha, sat, Ipfa, nfaE, gad, senB*
RA134	ST-205	B1	*gad, IpfA*
RA166	ST-131	B2	*iha, sat, cnf1, iss, gad, senB*
RA173	ST-131	B2	*iha, sat, Ipfa, nfaE, gad, ireA*
RA175	ST-131	B2	*iha, sat, iss, nfaE, gad, ireA*
RA176	ST-38	D	*gad, iss*
RA194	ST-617	A	*gad, iss*
RA195	ST-38	D	*gad, iss*
RA217	ST-44	A	*gad, iss*
RA218	ST-44	A	*gad, iss*
RA228	ST-38	D	*gad, iss*
RA246	ST-131	B2	*nfaE, Iha, sat, gad, iss*
RA256	ST-2852	B1	*gad, IpfA*

*gad*, Glutamate decarboxylase; *iss*, increased serum survival; *iha*, adherence protein; *sat*, secreted autotransporter toxin; *IpfA*, long polar fimbriae; *nfaE*, diffuse adherence fimbriae; *ireA*, siderophore receptor; *senB*, plasmid encoded enterotoxin; *cnf1*, cytotoxic necrotizing factor

## Discussion

The presented study identifies a high proportion of faecal carriage of ESBL-producing *E. coli* in the Mwanza community. The prevalence of 21 % in Igogo rural district is almost the same as that of 25 % obtained in *E. coli* clinical isolates in the same town [5]. Compared to a previous study [28] which investigated ESBL carriage in women and neonates admitted at Bugando Medical Centre, similar findings are observed with the magnitude of carriage observed in women. However, the carriage is significantly lower than that obtained in neonates. Our findings are within the range of the ESBL carriage reported in Africa which has been found to range from 10 % in Senegal to 31 % in Niger [20, 29–31]. The high carriage in Niger could be attributed to the fact that the population studied suffered from malnutrition with majority of individuals having previous antibiotic exposure.

As observed previously [18, 28], antibiotic exposure, history of admission, or increase in age were found to predict ESBL carriage on multivariate logistic regression analysis in our setting as well.

As in previous studies [16, 29], the *bla*_CTX-M-15_ was the commonest allele observed in the community in Mwanza, Tanzania. This allele has also been found to be the commonest allele in *E. coli* and *Klebsiella pneumoniae* isolates from Bugando Medical Centre [6, 32]. This could possibly be explained by contacts between individuals admitted to the hospital, and due to the fact that the Bugando Medical Centre is the only tertiary hospital in the region. Also environmental contamination by hospital sewages and transfer through the food-chain in the city such as the fish-consumption could play a significant role [33]. This is further supported by the fact that, as in hospital *E. coli* isolates from a previous study [32], ST-131 was commonly found in the community. However, more studies are needed to establish the transmission pathways especially the role of food-chain and environment in the persistence and spread of ESBL isolates in the city.

Though marginally significant on univariate analysis, people from Igogo were 1.9 times more likely to carry ESBL isolates than those from Kirumba. The population of these two rural districts are almost equal. However, Igogo rural district is nearer to the Bugando Medical Centre and has more industrial and garage activities than Kirumba. These could contribute to more environmental contamination that might lead to bacteria being resistant to various metals and chemicals. In addition, the population per pharmacy/medicine shops is low at Igogo rural district as compared to that in Kirumba rural district; this might contribute to an easy accessibility to antibiotics and more environmental contamination. More research to explore human’s activities, environmental contamination and the role of antibiotic usage are needed. A geographical factor worth studying regarding the influence on ESBL carriage is the fact that in all rural communities studied a majority of the population lives in the hills, and use latrines that are not connected to city sewage.

As compared to hospital isolates [5, 32], *E. coli* isolated in the community were more resistant to gentamicin, sulphamethoxazole/trimethoprim and tetracycline. These results necessitate an urgent review whether these antibiotics are of value for empirical treatment of infections caused by *E. coli* such as urinary tract infections.

This study further confirms the role of IncF plasmids with multiple resistance genes to be responsible in transmitting resistance genes among Enterobacteriaceae isolates [32, 34]. An important finding in this study is the detection of IncY plasmids in 20 % of isolates carrying quinolone resistance gene (*qnrS1*) and aminoglycosides resistance genes (*aadA5*, *strA* and *strB*) in addition to *bla*_CTX-M-15_. Similar plasmids have been recently detected in Nigeria among healthy pregnant women [35]. In addition, 57 % (13/25) of the isolates displayed a chromosomally located CTX-M-15, thereby enabling a vertical transfer of the resistance. The findings underscore the importance to investigate the epidemiology of ESBL-producing bacteria on the African continent to ascertain transmission pathways and factors associated to the persistence.

## Conclusion

High ESBL carriage, especially of *bla*_CTX-M-15_, is observed in the community among *Escherichia coli* in Mwanza city. Predictors for ESBL carriage are the use of antibiotic, history of hospital admission and increase in age. This information is useful for introducing pre-admission screening, planning empirical treatment by identifying high risk ESBL patients and adapting empirical treatment of infections towards covering ESBL isolates in patients with urosepsis. The findings that hospital clones and plasmids are also in the community isolates require more studies using a One Health approach to determine the role of human’s activities in relation to the persistence, circulation, spread and transmission of CTX-M-15-producing *E. coli* strains in Mwanza city in order to provide appropriate recommendations to control this public health threat.

## Ethical considerations

The protocol to perform this study was approved by Catholic University of Health and Allied Sciences and Bugando Medical Centre (CUHAS/BMC) ethics review board (CREC/043/2014). In addition, all participants signed written informed consent for participation in the study. Where participants were children, a parent or guardian signed the consent.

## Availability of data and materials

The raw data of the 25 sequenced *E. coli* are available at the European Nucleotide Archive (ENA) under the project number PRJEB12376.

## Abbreviations

BMC: Bugando Medical Centre
CC: clonal complex
CTX: cefotaxime
CUHAS: Catholic University of Health and Allied Sciences
ESBL: extended spectrum beta-lactamases
gad: glutamate decarboxylase
iha: adherence protein
Inc: incompatibility
IQR: interquartile range
iss: increased serum survival
OR: odd ratio
PCR: polymerase chain reaction
sat: secreted autotransporter
SHV: sulfhydryl variable
ST: sequence type
Str: streptomycin resistant
TEM: temoniera
Tet: tetracycline resistant
WGS: whole genome sequence
